# Linking tumor immune infiltrate and systemic immune mediators to treatment response and prognosis in advanced cervical cancer

**DOI:** 10.1038/s41598-023-49441-2

**Published:** 2023-12-19

**Authors:** Patrícia Rocha Martins, Kátia Luciano Pereira Morais, Nayane Alves de Lima Galdino, Adriana Jacauna, Sálua O. C. Paula, Wagner C. S. Magalhães, Luciana W. Zuccherato, Larissa S. Campos, Paulo Guilherme O. Salles, Kenneth J. Gollob

**Affiliations:** 1 Pathology Department, Universidade Federal de Minas Gerais, Belo Horizonte, MG Brazil; 2 Instituto Mário Penna, Belo Horizonte, MG Brazil; 3 Translational Immuno-Oncology Lab, Education and Research Institute, Hospital Israelita Albert Einstein, São Paulo, SP Brazil; 4 Center for Research in Immuno-Oncology (CRIO), Hospital Israelita Albert Einstein, São Paulo, SP Brazil; 5 CCATES – Centro Colaborador do SUS: Avaliação de Tecnologias e Excelencia em Saude, UFMG, Belo Horizonte, Brazil; 6 Pontificia Universidade Catolica de Minas Gerais, Belo Horizonte, Brazil; 7 Department of Biochemistry and Immunology, Universidade Federal de Minas Gerais, Belo Horizonte, MG Brazil; 8 Center for Research in Immuno-Oncology (CRIO), Translational Immuno-Oncology Laboratory, Hospital Israelita Albert Einstein, Av. Albert Einstein, São Paulo, SP 62705652-900 Brazil

**Keywords:** Translational immunology, Tumour immunology, Gynaecological cancer

## Abstract

Cervical cancer (CC) poses a significant burden on individuals in developing regions, exhibiting heterogeneous responses to standard chemoradiation therapy, and contributing to substantial mortality rates. Unraveling host immune dynamics holds promise for innovative therapies and discovery of clinically relevant biomarkers. We studied prospectively locally advanced CC patients pre-treatment, stratifying them as responders (R) or non-responders (NR). R patients had increased tumor-infiltrating lymphocytes (TILs), while NR patients showed elevated PD-1 scores, CD8+ and PD-L2+ TILs, and PD-L1 immune reactivity. NR patients exhibited higher systemic soluble mediators correlating with TIL immune markers. R patients demonstrated functional polarization of CD4 T cells (Th1, Th2, Th17, and Treg), while CD8+ T cells and CD68+ macrophages predominated in the NR group. Receiver operating characteristic analysis identified potential CC response predictors, including PD-L1-immunoreactive (IR) area, PD-L2, CD8, FGF-basic, IL-7, IL-8, IL-12p40, IL-15, and TNF-alpha. Dysfunctional TILs and imbalanced immune mediators contribute to therapeutic insufficiency, shedding light on local and systemic immune interplay. Our study informs immunological signatures for treatment prediction and CC prognosis.

## Introduction

Cervical cancer (CC) is the fourth most common cancer among women worldwide in terms of both frequency and mortality^1^. The most significant risk factor for CC is persistent human papillomavirus (HPV) infection, which has an overall prevalence of 25% in the cervix in Brazil, higher than the prevalence estimated for the global (11.7%) and Latin American (16%) populations^2,3^.

Despite being preventable, the high prevalence of HPV in the world population remains a major public health concern. CC patients exhibit variable response to concurrent chemoradiotherapy and high recurrence rates, resulting in a 5-year survival rate ranging from 30 to 80%^4^. Furthermore, approximately 90% of CC-related deaths occur in women with limited access to prevention, screening, and early treatment programs^5^. Two-thirds of CC patients present advanced stages (IB2-IVA) according to the International Federation of Gynecology and Obstetrics (FIGO) classification.

Although most patients are treated using the same protocols, each patient exhibits an inconsistent overall response and prognosis, primarily due to the heterogeneity of the tumor and the patient's immune system^6^. Therefore, identifying strategies that facilitate the identification of future responders (R) or non-responders (NR) to chemoradiotherapy could result in more effective treatment and reduce the costs to the health system^7^. Increasing evidence suggests that host inflammatory responses play a vital role in the development and progression of cancers^8^. Cancer is associated with not only localized inflammation but also dysregulation of the overall systemic immune response^9^. Inflammation is an essential factor associated with the development, progression, and potential metastasis of CC^10^.

A compromised immune system increases the risk of HPV infection and reduces the successful response rate to treatment^11,12^. Cancer cells, resident tissue cells, and immune cells release anti- and pro-inflammatory cytokines that form the tumor microenvironment (TME), and subsequently lead to a better or poor response to therapies depending on this balance^13^. The interaction between antigen-presenting cells and T cells mediates malignant cell recognition^14^, and higher rates of tumor-infiltrated lymphocytes (TILs) are associated with better prognosis in many solid tumors^15,16^. However, cancer cells express suppression signals on the cell surface to inhibit the immune function of activated T cells and decrease tumor immune clearance. These negative immune pathways are described as immune checkpoints^17^. The binding between programmed death 1 protein (PD-1) expressed in T cells and their ligands 1 and 2 (PD-L1 and PD-L2) in cancer cells promotes exhaustion and T cell death^18^.

Ongoing research has tested the inhibition of immune checkpoints and provided alternatives to traditional CC treatment modalities^19,20^. Many studies have defined "hot" and "cold" tumors based on three basic immune profiles (inflamed, excluded, and desert-immune phenotype) that correlate with distinct responses to immunotherapy^21–23^. Inflamed tumors, also known as "T-cell-inflamed," exhibit anti-tumor T-cell aggregation before treatment, while non-inflamed or cold tumors exhibit a lack of T-cell aggregation^24^. Furthermore, changes in local and systemic cytokine levels can reflect the immune response of lymphocyte populations induced by cancer cells^25^. CC is associated with inflammation at the site of the lesion, as well as dysregulation of the overall systemic immune response^9,10,26^.

Therefore, identifying specific subpopulations of TILs and their location within the tumor microenvironment (intra-tumoral or stromal) along with the systemic immune profile of CC could serve as prognostic indicators and shed light on immune mechanisms behind response to therapy. In this study, we aimed to investigate a diverse panel of systemic soluble immune mediators and tissue immune markers, before therapy began, in locally advanced CC patients undergoing chemoradiotherapy.

## Results

### Clinicopathological characteristics and patient survival

A total of 163 women with locally advanced cervical cancer (CC) were enrolled in this prospective study, however 73 were included in the final analysis. 90 patients were excluded from the study due to; (1) a failure to complete all sessions of the paclitaxel + cisplatin chemotherapy concurrent with radiotherapy and brachytherapy (49 patients), (2) a loss of follow-up (16 patients), and (3) they were actively in treatment at the time of analysis (25 patients). The clinicopathological and immune response profiles, as well as patient survival was fully analyzed in the 73 cases. The cases were divided into two groups based on response to treatment: NR (n = 34) and R (n = 39). Most patients in both groups had bilateral parametrial and vaginal involvement and were classified as stage IIB/IIIB with moderate or poorly differentiated histology grades. There were no significant differences in most clinical features between the groups, except for lymph node invasion and metastasis.

**Figure 1:**
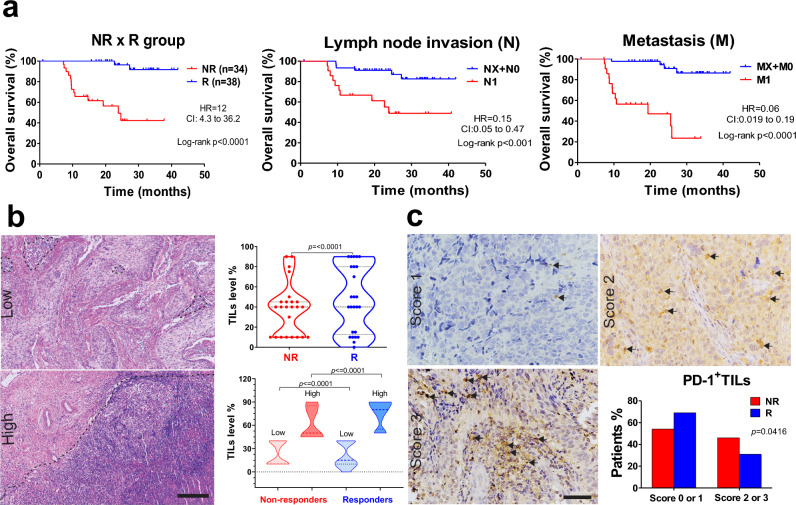
CC patients that respond to chemoradiotherapy exhibit a high survival percentage, high immune cell infiltration, and lower PD-1 score than non-responder patients. (**a**) Kaplan–Meier survival curves highlight the higher percentage of overall survival in R than NR patients (NR, n = 34 and R, n = 38). Patients with lymph node invasion (N1; NR, n = 48 and R, n = 24) and metastasis (M1, NR, n = 49 and R, n = 23) displayed lower survival. (**b**) Representative images of low and high stromal TILs stained by H&E. Black outlining separates the TILs area considered. Scale bar = 200 μm. Morphometric analysis in H&E-stained slides illustrated by violin plot showed a higher TILs percentage in the R than NR group (Wilcoxon Signed Rank Test, *p* =  ≤ 0.0001; NR, n = 24 and R, n = 25) and when segregated by low and high stromal TILs (Mann–Whitney test, *p* =  ≤ 0.0001; NR low, n = 15; NR high, n = 9; R low, n = 13 and R high, n = 12). (**c**) Images of PD-1^+^ stromal TILs IHC-stained (arrows) in low (1), medium (2), and high (3) scores. Scale bar = 50 μm. The bar graph represents the percentages of NR and R patients when comparing the scores of 0 or 1 and 2 or 3 (Fischer’s exact test, RR = 0.7349; OR = 0.52, 95% CI 0.2958–0.9404, p = 0.0416; NR, n = 24 and R, n = 26). CC, Cervical Cancer; NR, non-responder (Red) patients; R, responder (Blue) patients; TILs, Tumor-infiltrating lymphocytes; H&E, Hematoxylin & Eosin; HR, Hazard Ratio; RR, Relative risk; OD, Odds ratio; CI, Confidence interval; N, Local lymph node invasion at diagnosis (NX, not evaluated; N0, absent; N1, present); M, Distant metastasis at diagnosis (MX, not evaluated; M0, absent; M1, present).

Analysis showed that lymph node invasion and metastasis were significant positive prognostic factors in the R patients (OR, 0.19, 95% CI 0.067–0.56, p = 0.0027 and OR 0.051, 95% CI 0.013–0.20, p < 0.0001, Table 1). Kaplan–Meier curves were used to assess overall survival (OS) in the cohort of 73 patients. The NR patients showed poorer survival outcomes, while the R group showed 91.7% survival, as expected. Patients with lymph node invasion (N1) and metastasis (M1) showed significantly lower survival percentages (Fig. 1a).

**Table 1:** Cervical cancer cohort clinical data.

Clinical data					OR	CI
		n (%)		p-value		
		NR	R			
	n	34	39	0.26^a^		
Age		47.2 ± 2.4	50.5 ± 2.1			
Diagnosis	SCC	32 (94.1)	35 (89.7)	0.67^b^	1.829	0.3133 to 10.67
	ICA	2 (5.9)	4 (10.30)			
	I-B2	1 (2.9)	–			
FIGO stage	II-A	1 (2.9)	2 (5.1)	1.0^c^		
	II-B	14 (44.1)	20 (51.3)			
	III-A	NA	1 (2.6)			
	III-B	18 (50)	15 (38.5)			
	NA	–	1 (2.6)			
Histologic grade	I	–	1 (2.6)	0.70^c^		
	II	17 (50)	16 (41.0)			
	III	11 (32.4)	16 (41.0)			
	NA	6 (17.6)	6 (15.4)			
Parametrial involvement	Free	2 (5.9)	1 (2.6)	0.5^c^		
	Unilateral	6 (17.6)	11 (28.2)			
	Bilateral	26 (76.5)	25 (64.1)			
	NA	–	2 (5.3)			
Vaginal involvement	Yes	31 (91.2)	36 (92.3)	1.0^c^		
	No	2 (5.9)	2 (5.1)			
	NA	1 (2.9)	1 (2.6)			
Lymph node invasion	Yes	18 (53.0)	7 (18.0)	**0.0027**^**b**^	0.1944	0.06739 to 0.5611
	No	16 (47.0)	32 (82.0)			
Metastasis	Yes	21 (61.8)	3 (7.7)	**0.0001**^**b**^	0.05159	0.01316 to 0.2022
	No	13 (38.2)	36 (92.3)			
Tumor size (cm)	4 ≤	2 (5.9)	7 (17.9)	0.14^c^		
	4 >	30 (88.2)	27 (69.2)			
	NA	2 (5.9)	5 (12.8)			

SCC, squamous cell carcinoma; ICA, invasive cervical adenocarcinoma NR, non-responders, R, responders; NA, not available; OR, odds ratio; CI, 95% confidence interval.

^a^Student’s t-test.

^b^Fisher’s exact test.

^c^Chi-square test. P values < 0.05 are marked in bold.

### Overall tumor immune profile in R and NR cervical cancer patients

Here we aimed to delineate the immune profile of CC patients by assessing the intensity of immune cell infiltration densities through Hematoxylin & Eosin (H&E)-stained slides and immunohistochemistry (IHC). Patients were classified into low or high-expression groups based on individualized cut-off values (Suppl. Table 1). Representative images of tumor sections with minimal or high immune cell infiltration are illustrated in Fig. 1b. R patients showed increased inflammatory infiltrate, even when displaying low and high percentages of stromal TILs (Fig. 1b). Assessment of PD-1+ TILs expression (Fig. 1c) showed that patients with scores of 0 or 1  had a relative risk of 0.7349 to reach NR outcome and an odds ratio of 0.52 in comparison to those with scores of 2 or 3 (95% CI 0.2958–0.9404, p = 0.04; Fig. 1c).

**Figure 2:**
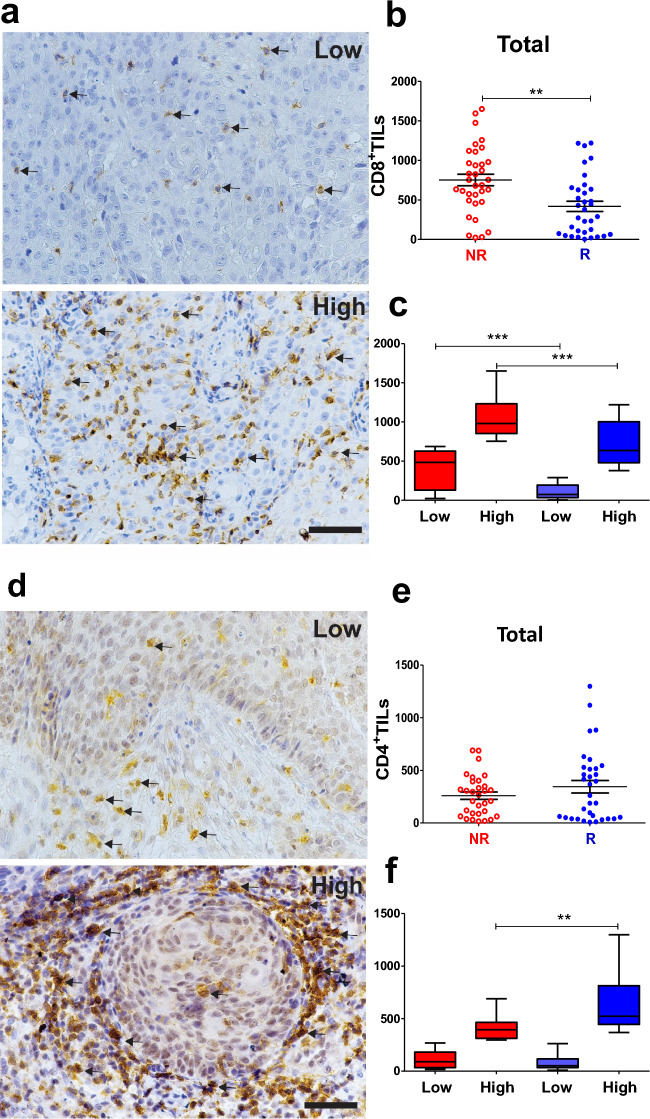
Non-responder CC patients exhibited elevated numbers of CD8^+^ TILs. Representative IHC images (**a** and **d**) of brown stained immune cells (+) indicating the expression of CD8^+^ (**a**) and CD4^+^ (**d**) TILs in human CC taken before treatment began at the time of diagnosis. The arrows indicate lymphocytes at high magnification (400x). Scale bar = 50 μm. (**b** and **e**) Morphometric analysis showed NR with a higher number of CD8^+^ TILs than in R patients (**b**, NR, n = 33 and R, n = 34) and no difference in the number of CD4^+^ TILs (**e**, NR, n = 32 and R, n = 33). (**c**) Number of CD8^+^ TILs plotted in low and high cell densities showed a higher number of TILs in NR than R group (low NR vs. R and high NR vs. R; NR low, n = 16; NR high, n = 17; R low, n = 17 and R high, n = 17). (**f)** Higher number of CD4^+^ TILs in the total sum of R (high R vs. NR; NR low, n = 16; NR high, n = 16; R low, n = 17 and R high, n = 16). Statistical differences are indicated by asterisks (*) *p* ≤ 0.05, (**) *p* ≤ 0.01, and (***) *p* ≤ 0.001. The *p* values were calculated using the Mann–Whitney test. The graphs show the median and standard error. CC, Cervical Cancer; NR, non-responder (Red) patients; R, responder (Blue) patients.

**Figure 3:**
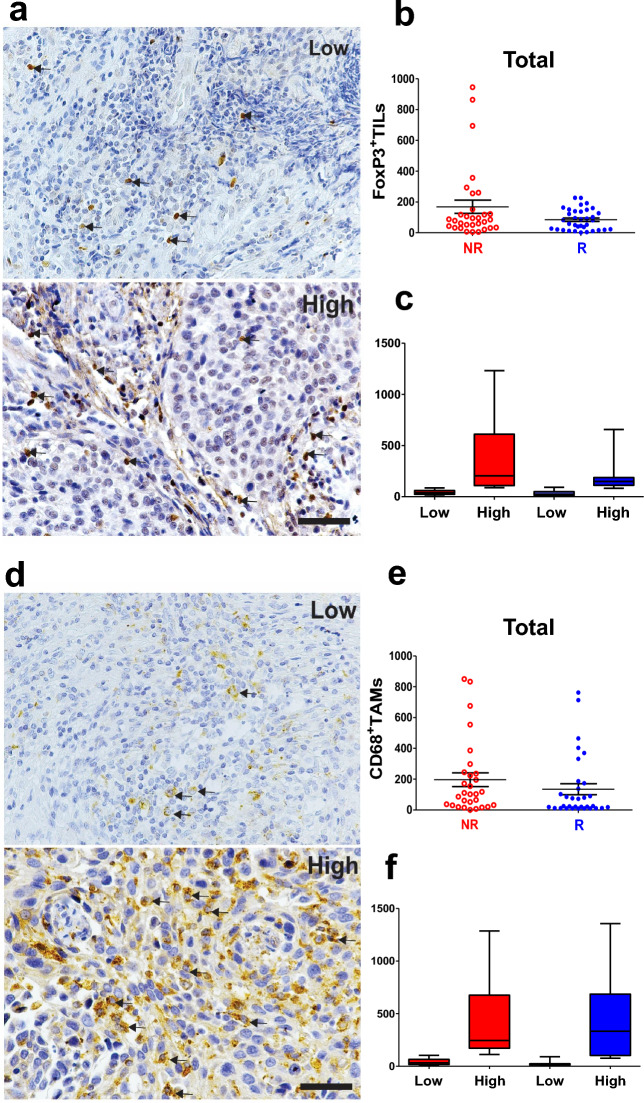
Total FoxP3^+^ TILs and CD68^+^ TAMs expression in  CC patients. Representative IHC images of low and high FoxP3^+^ TILs (**a**) and CD68^+^ TAMs (**d**) are indicated by arrows. Scale bar = 50 μm. Morphometric analysis did not show differences in total FoxP3^+^ TILs quantification between the groups (**b**, NR, n = 32 and R, n = 36) and (**c**, NR low, n = 16; NR high, n = 16; R low, n = 19 and R high, n = 17). (**e**) No difference was observed in the number of total CD68^+^ TAMs between the groups (NR, n = 30 and R, n = 34) and (**f**, NR low, n = 15; NR high, n = 15; R low, n = 19 and R high, n = 15), considering *p* ≤ 0.05. The *p* values were calculated using the Mann–Whitney test. The graphs show the median and standard error. CC, cervical cancer; NR, non-responder (Red) patients; R, responder (Blue) patients.

**Figure 4:**
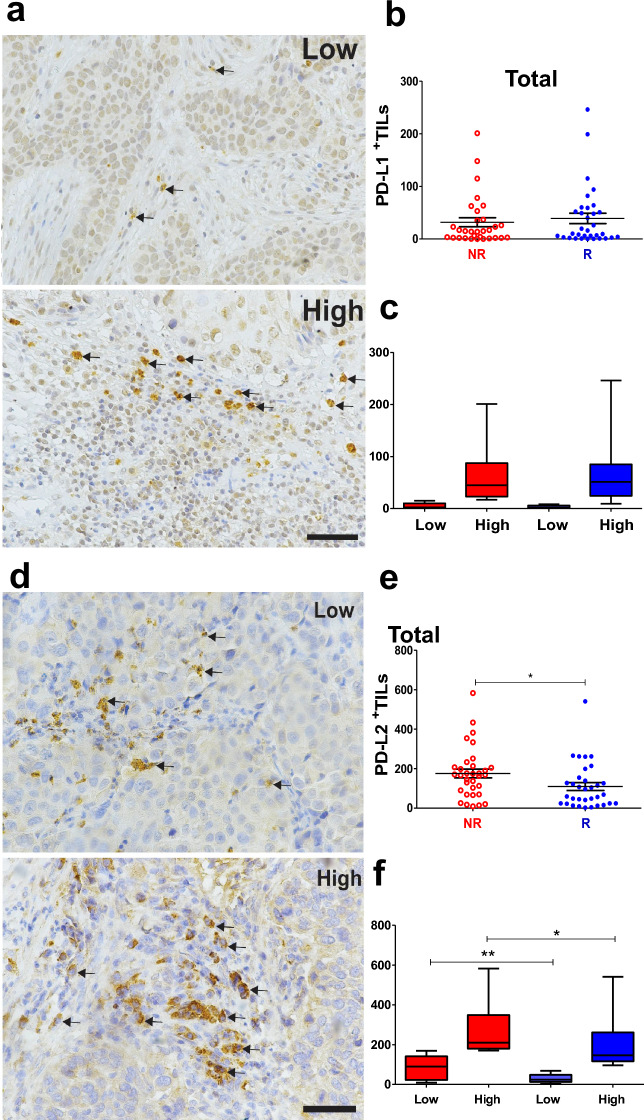
Non-responder CC patients presented a high number of PD-L2^+^TILs. Representative IHC images of low and high PD-L1^+^ TILs (**a**) and PD-L2^+^ TILs (**d**) are indicated by arrows. Scale bar = 50 μm. Morphometric analysis did not show differences in PD-L1^+^ TILs quantification between the groups (**b**, NR n = 31; R n = 34) and (**c**, NR low, n = 17; NR high, n = 14; R low, n = 16 and R high, n = 18), the. (**e**) NR patients presented higher number of PD-L2^+^ TILs in total sum than R patients (NR n = 33; R n = 34), considering *p* ≤ 0.05. (**f**) Quantification analysis showed a higher median of PD-L2^+^ TILs in NR, when comparing low cell densities in the total sum and stromal region and comparing the high cell densities of PD-L2 in NR vs R patients (low NR vs. R and high NR vs. R; NR low, n = 17; NR high, n = 16; R low, n = 17 and R high, n = 17). Statistical differences are indicated by asterisks (*) *p* ≤ 0.05 and (**) *p* ≤ 0.01. The *p* values were calculated using the Mann–Whitney test. The graphs show the median and standard error. CC, Cervical Cancer; NR, non-responder (Red) patients; R, responder (Blue) patients.

Next, we investigated TIL immune markers, and higher numbers of CD8+ TILs were found in NR CC patients, independent of the evaluated compartment (Fig. 2b and Suppl. Fig. 1b). The stratification of stromal TILs revealed heterogeneity in both R and NR groups, with low and high expression of all immune markers (Figs. 2, 3, 4a,d and c, f; Suppl. Figs. 1–3c, g).

CD8+ TILs were elevated in NR patients, classified as low or high expression (Fig. 2c and Suppl. Fig. 1c). Importantly, stromal FoxP3+ (Suppl. Fig. 2c) and stromal CD68+ TAMs (Suppl. Fig. 2g) were elevated in NR patients when compared to low NR vs. R, while total FoxP3+ and total CD68+ TAMs did not differ between the groups (Fig. 3). In contrast, R patients showed more infiltration of CD4+ TILs in the total sum (Fig. 2e) and intra-tumoral region (Suppl. Fig. 1g) when comparing high levels in R vs. high in NR. These results indicate a distinct immune response pattern in NR compared to R patients, with a bias for potentially suppressive populations in the NR compared to the R group (FoxP3+ and CD68+ TAMs).

Regarding OS, differences were observed only between low NR vs. low R and high NR vs. high R for the great majority of immune markers. No difference was observed between low vs. high in the same group for these markers (R or NR, Suppl. 1–3d and h). However, the exception was the stromal PD-L2+ TILs, which displayed significantly worse OS for the high expression group as compared to the low group (*p* < 0.04, HR 0.35, 95% C.I 0.12–0.18, Suppl. 3i). All the details concerning TILs, both total sum or by compartments, can be found in Figs. 2, 3, 4b,e, and Suppl. 1–3b,f.

### NR patients exhibited elevated levels of PD-L1+ neoplastic cells and PD-L2+ TILs, which were associated with poorer survival outcomes

The prognostic value of PD-L1 and PD-L2 checkpoints in CC is still being debated^27^, and thus we examined TILs (Fig. 4 and Suppl. Fig. 3) and the total area of cancer cells (Table 2) expressing these molecules. TILs expressing PD-L1 and PD-L2 were more commonly observed in the stromal than intra-tumoral regions (Fig. 4a and d, Suppl. Fig. 3a and e). However, no difference was observed in the number of TILs expressing PD-L1 between the R and NR groups (Fig. 4b and Suppl. Fig. 3b), nor in the low and high PD-L1+ TILs densities (Fig. 4c and Suppl. Fig. 3c). In contrast, when considering all IR areas (stromal and intra-tumoral regions), a higher PD-L1 total area was found in NR (Table 2), indicating that neoplastic CCs express more PD-L1 than TILs. Additionally, PD-L2+ TILs were more frequently observed in NR than R patients (Fig. 4e and Suppl. Fig. 3f). When comparing the low and high cell densities between the two groups, the low density of PD-L2+ TILs in the total sum and stromal TILs was higher in NR than R (Fig. 4f and Suppl. Fig. 3g). The same trend was observed when comparing the high density of PD-L2+ TILs in the stromal region of NR vs. R (Suppl. Fig. 3g). The PD-L2-IR area did not differ between the groups, indicating that TILs in CC express more PD-L2 than the tumor cells, in contrast to PD-L1 expression (Table 2).

**Table 2:** Immunoreactive area of PD-L1 and PD-L2 in CC of NR and R patients.

		Total area		Stromal region		Intra-tumoral region	
PD-L1 IR area							
Non-responders	23	7376 ± 1457	**0.0325**	1030 ± 265	0.2076	4972 ± 1468	**P < 0.0001**
Responders	24	2787 ± 1008		2042 ± 912		204 ± 190	
PD-L2 IR area							
Non-responders	21	5228 ± 1495	0.6592	678 ± 369	0.9473	3383 ± 1257	0.3430
Responders	25	3832 ± 1396		556 ± 196		3294 ± 1265	

Mann–Whitney test. Median (MD) and Std. Error (SE). P values < 0.05 are marked in bold.

### NR patients display higher levels of soluble mediators than responders.

**Figure 5:**
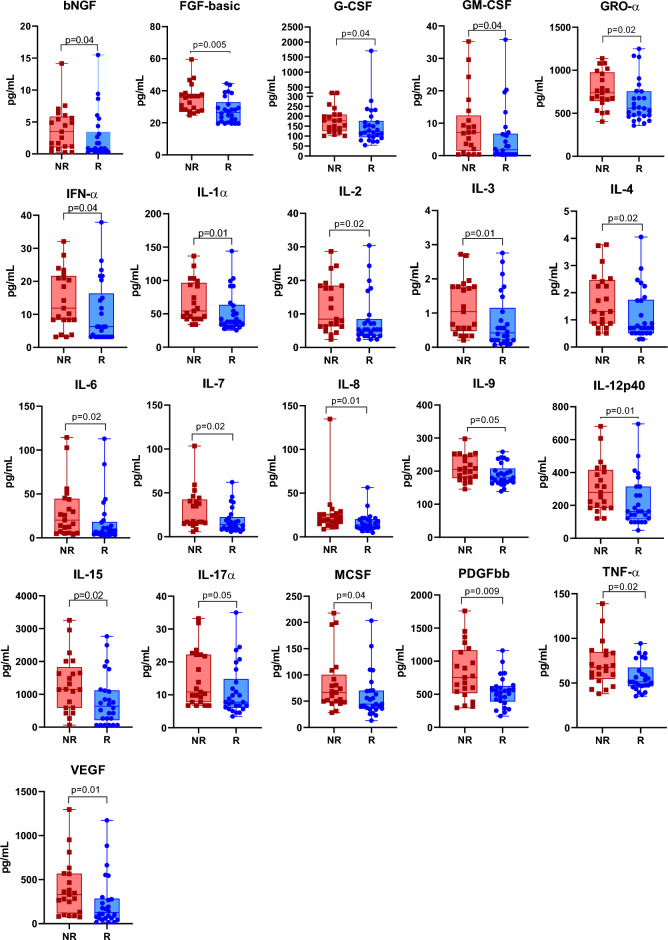
Higher levels of soluble immune mediators in CC non-responder patients. Soluble immune mediators were measured in the plasma of patients by using the Bio-Plex Pro Human Cytokine Screening Panel, 48-Plex according to the manufacturer's instructions test. CC, Cervical Cancer; NR, non-responder (n = 21); R, responder (n = 26). Analytes with p-values ≤ 0.05 after the Mann–Whitney test are shown.

Cytokines and chemokines mediate local and systemic immune responses^28^, which can either promote tumor growth or stimulate antitumor immune responses. The clinical significance of these factors has been investigated over the years^29–31^. Thereby, to investigate the possible association between soluble immune mediators with CC patients’ response to therapy, we used the Bio-Plex® 200 system to analyze 48 soluble mediators and found higher levels of 21 analytes in the plasma of NR patients than R patients. Levels of the chemokines GRO-α, IL-8, IL-7 were increased in NR patients when compared to R patients. Similarly, the concentration of growth factors β-NGF, VEGF, FGF basic, G-CSF, GM-CSF, IL-3, M-CSF, PDGF-BB were higher in the NR patients, as well as proinflammatory cytokines IFN-α2, IL-1α, IL-2, IL-6, IL-12p40, IL-15, IL-17, TNF-α and regulatory IL-4 and IL-9 cytokines (Fig. 5). These findings suggest that CC patients who will not respond to therapy display higher levels of key immunomodulatory soluble mediators.

### NR patients exhibit high tumor microenvironment PD-L1, associated with decreased systemic cytokines/chemokines, while R patients demonstrate a well-regulated association between TILs, TAMs, and soluble mediators

**Figure 6:**
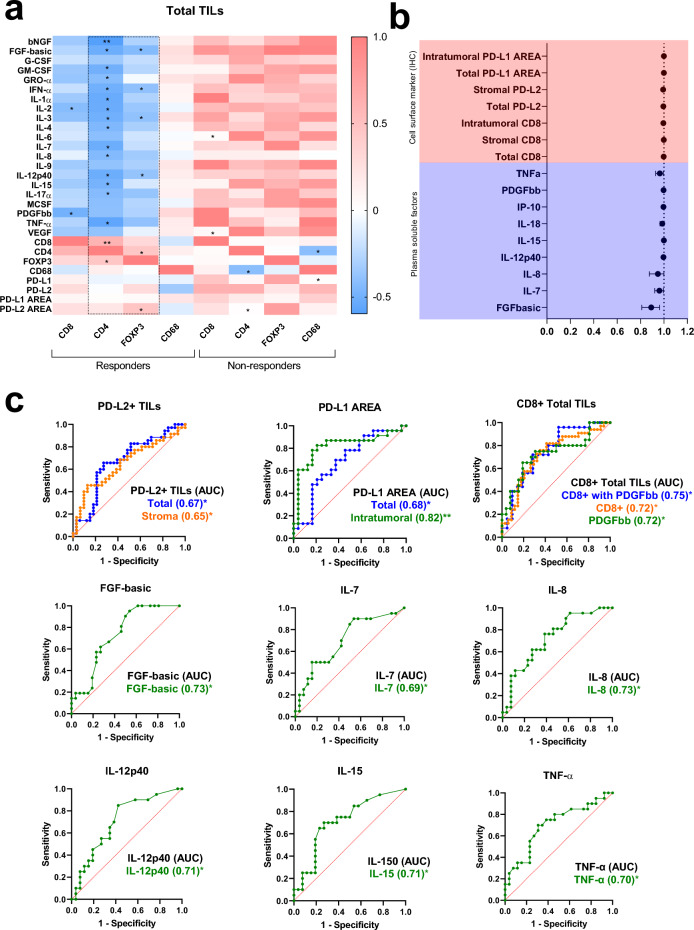
Associations between systemic immune mediators and TME immune indicators reveal distinct immune mechanisms in responder versus non-responder patients. (**a**) Correlation matrix between systemic soluble immune mediators and TIL subpopulations. All statistical analyses were performed with Spearman’s correlation Test. (**b**) Forest plots indicating independent predictors of treatment response based on systemic soluble immune mediators and TILs markers or PD-L1-IR area in CC patients. Odds Ratio (OR) and 95% confidence intervals (95% CI) were calculated using a logistic regression model. (**c**) The capacity of systemic soluble immune mediators and TILs markers or PD-L1-IR area before treatment began to distinguish R vs. NR CC patients was evaluated by receiver operating characteristic (ROC) curves. The Area Under the Curve (AUC) was calculated for all parameters. CC, Cervical Cancer; NR, Non-responder (n = 21); R, Responder (n = 26); TME, tumor microenvironment. In all graphics: *p ≤ 0.05, **p ≤ 0.001.

We next conducted further investigation to evaluate immune networks through the association between systemic cytokine levels and the TME identified by IHC. Interestingly, we found a positive correlation between the number of CD4+ TILs and FoxP3 only in the R group (Fig. 6A), suggesting the involvement of Treg cells. This finding was reinforced by the negative correlation of both CD4+ and FoxP3+ TILs with FGF basic, IL-3, IFNα, and IL-12p40, all of which were only observed in the R group (Fig. 6a). Furthermore, we observed a positive correlation between FoxP3+ TILs and the PD-L2-IR area, which has been previously shown to regulate metabolic pathways involved in the suppressive functions of Treg cells. Notably, this correlation seemed to be more prominent in the intra-tumoral compartment, which has generated interest in recent studies due to the potential suppression mechanisms used by intra-tumoral Tregs, facilitated by various adaptations in the TME^32–34^.

Moreover, we highlighted a potential role of CD4+ TILs in the R group by observing negative correlations with eleven plasma soluble mediators, including those associated with Th1 (IL-2, IL-12p40, TNF-α), Th2 (IL-4), and Th17 (IL-17) profiles. The preferential distribution of these TILs in the intra-tumoral and stromal regions seems to be crucial in this mechanism, as most correlations were lost when the intra-tumoral region was analyzed separately, except for IL-17 (Suppl. Figs. 4, 5, and 6).

**Figure 7:**
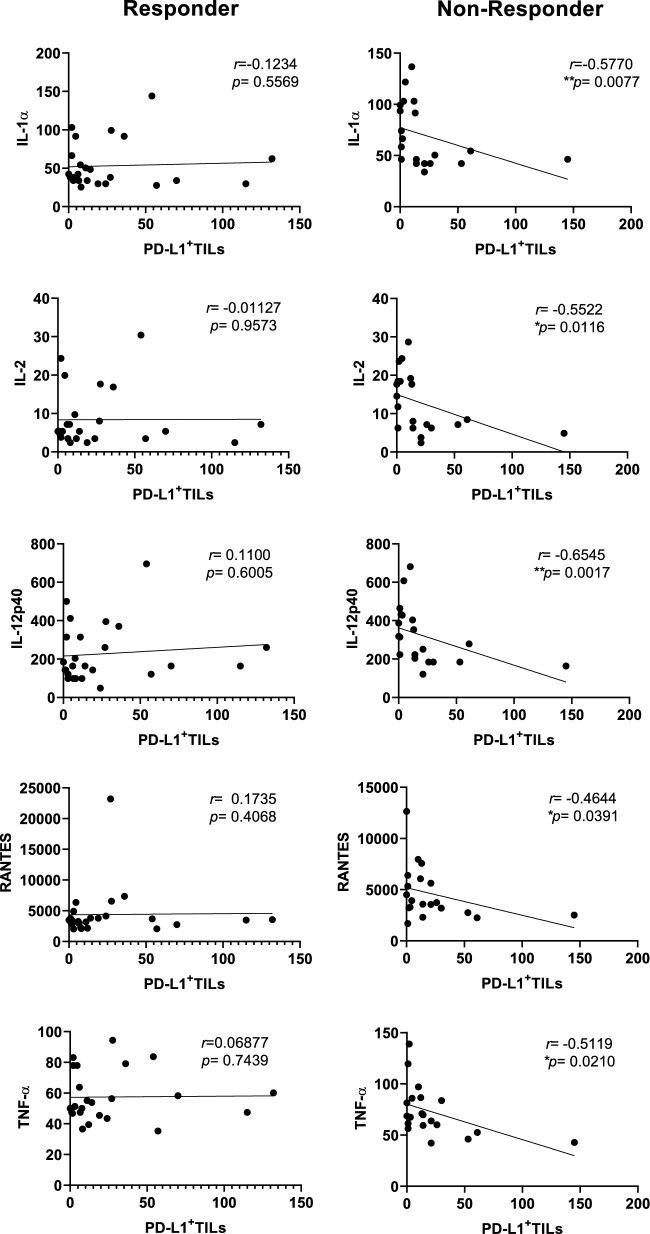
Stromal PD-L1 expression is associated with a downregulation of systemic anti-tumor response promoting cytokines and chemokines in NR patients. Correlation between stromal PD-L1^+^TILs and soluble immune mediators in CC patients. CC, Cervical Cancer; NR, non-responder (n = 21); R, responder (n = 26). All statistical analyses were performed with the Spearman’s correlation Test, (*) *p* ≤ 0.05, (**) *p* ≤ 0.01. Immune mediators are expressed in pg/ml.

In contrast, in the NR group, the correlation results were different. CD68+ TAMs were negatively correlated with CD4+ and PD-L1+ TILs (Fig. 6), which suggests a process of recruitment and activation of immunosuppressive or unconventional macrophages. Additionally, we observed a positive correlation between CD8 TILs and VEGF and IL-6, indicating a potential link between T cell infiltration and differentiation, as well as vascularization in the NR group, which appears to be more predominant in the stromal region. One striking negative correlation in the NR group was seen between TME PD-L1 positive cells (stromal region) and many systemic cytokines and chemokines known to be important for inducing an anti-tumor response via tissue migration and inflammation including IL-1a, IL-1b, IL-12p40, RANTES, SDF1a, TNF-a, TNF-b and others (Fig. 7 and Suppl. Figs. 4, 5 and 6). This negative correlation between higher TME PD-L1 and lower systemic cytokines/chemokines is lacking in the R group across all regions (total, stromal and intra-tumoral).

We also conducted complete correlation matrices with all 48 cytokines and cell markers (total, stromal, and intra-tumoral) in both groups (Suppl. Figs. 4, 5, and 6, respectively) the above-mentioned networks can be seen in the context of all molecules investigated.

### Soluble mediators and TILs predict clinical outcomes in cervical cancer

To evaluate the ability of soluble mediators and cell markers to predict treatment response in locally advanced CC to chemoradiotherapy, we first conducted a logistic regression analysis, followed by receiver operating characteristic (ROC) curve analysis. The individual predictors identified were PD-L1-IR area (total and intra-tumoral), PD-L2 TILs (total and stromal), CD8, FGFbasic, IL-7, IL-8, IL-12p40, IL-15, IL-18, IP-10, PDGFbb, and TNF-α (Fig. 6b). Also, the ability of CD8+ TILs to identify R vs. NR patients was similar to that seen in combination with PDGFbb (Fig. 6c).

ROC analysis revealed that the PD-L1-IR area was an excellent predictor, with an area under the curve (AUC) of 0.82. For soluble mediators, the AUC range was between 0.69 and 0.73, and combining them with cell surface markers resulted in similar values. For example, the AUC was 0.72 for CD8 and PDGFbb, while in the multivariate ROC curves (CD8 with PDGFbb), the AUC was 0.75 (Fig. 6c). However, IP-10 and IL-18, which were identified as independent predictors by logistic regression, had poor AUC scores of 0.59 and 0.61, respectively (data not shown).

## Discussion

Dysregulated immune function is closely linked to tumor initiation, tumor progression, and metastases in CC as well as other tumor types. This includes both local cellular immunity responses and systemic soluble mediator networks^35–38^. Furthermore, increasing evidence supports the evaluation of cell markers and immune mediators as predictors of response to treatment or mechanisms of disease development^39–43^.

In this study, we investigated tissue immune markers in CC patients prior to treatment, distinguishing between those who respond or not to chemoradiotherapy treatment. Overall, the morphological evaluation of immune cell composition in the TME illustrated the local immune functional status of these patients. Specifically, the R patients were characterized by a higher percentage of TILs indicating an inflamed profile. In agreement, other studies have shown that a high TILs percentage is a good prognostic factor in CC patients^42,44^. Additionally, the high PD-1 score associated with NR outcome (Fig. 1c) suggests the presence of dysfunctional TILs in these patients in an exhaustion state. Previous studies have emphasized the immune down-regulatory response of PD-1 expressing T cells^45^. Yin and colleagues demonstrated that CC patients treated with injections of TILs presented higher progression-free survival (PFS) and OS, while poor survival was observed when TILs high in CD8^+^PD-1^+^ cells were injected^46^.

The distinct immune functional status between our CC patient groups may also contribute to the OS rate, with NR patients showing  12 times higher risk of death compared to R patients. Moroever,  lymph node invasion and metastasis, were both associated with an increased mortality rate.

Furthermore, our data indicated that the NR patients had higher numbers of CD8+ and PD-L2+ TILs, as well as a higher PD-L1 IR area, suggesting a dysfunctional state of TILs. T cell–inflamed tumors are described by CD8+ T cell infiltration, chemokines that attract T cells, type I IFN activation, cytotoxic effector molecules, higher numbers of FoxP3+, and immune inhibitory pathways^24,47^. The events that occur to develop an antitumor response lead to T cell infiltration and an inflamed TME. However, the immune response is attenuated by tumor PD-L1/2 upregulation, leading to dysfunctional T cells^24^. Therefore, a similar process may occur in our patient cohort. Furthermore, PD-L2 levels measured before therapy began were higher in NR patients and were associated with worse OS. This is similar to other studies that observed higher PD-L2 expression in patients with high-grade cervical intraepithelial neoplasia, precancerous lesions^48^.

Moreover, our findings revealed that neoplastic cells in NR CC patients exhibited higher expression of PD-L1 than TILs, while the opposite is observed for PD-L2. Interestingly, PD-L2 expression in malignant cells differs among various types of tumors but not in immune cells^49^. In CC patients, as well as other tumors, there are variations in the expression of these markers in tumor and immune cells, with a significant number of samples showing PD-L2 expression in the absence of PD-L1^49,50^. Clinical response to immunotherapy is predicted by both immune checkpoints when the total IR area (tumor and immune cells) is combined, as shown in one study^50^. Another study showed that only when the expression in tumor and immune cells were combined, PD-L1 expression was associated with a response to pembrolizumab^51^.

Notably, here the density of stromal CD68+ TAMs was found to be elevated in NR patients, which is consistent with other studies^52,53^. Although here CD68+ TAM numbers did not present a difference in OS, a higher number of CD68+ TAMs in stromal regions was associated with tumor progression^54^ and poor prognosis^55^ in other studies.

Furthermore, our findings demonstrated that NR patients exhibited higher levels of several systemic soluble mediators before therapy began, indicating an imbalance in their production and hyperactivation of the immune system, which can lead to disease progression and therapy failure^56,57^. Many soluble mediators have a well-established role in cancer biology, as they recruit and reprogram other types of cells within the TME that support the pathological process^29,30,58^. Our results showed elevated plasma levels of FGF, IL-6, IL-8, M-CSF, VEGF, TNF-alpha, and IL-17a, molecules previously associated with larger tumor size, metastasis, and poor prognosis^31,59–62^, as well as persistent HPV infection in older women with evidence of an immune deficit^63,64^. Interestingly, γ chain cytokine family members were increased before therapy began in NR patients, such as IL-2, IL-4, IL-7, IL-9, IL-15^65–68^. These cytokines mediate biological actions on a range of immune cells and play pivotal roles in the control of lymphocyte activation and differentiation. In addition, IFN-α2 involved in the regulation of T-cell function and differentiation was also increased in the NR group^69,70^. Also, soluble mediators GM-CSF, G-CSF, IL-3, and IL-1a are important for development, recruitment, and responses mediated by myeloid cells^71–74^, and both growth factors PDGFbb and bNGF involved in immune system regulation^75–77^ were differentially expressed between NR and R patients. Overall, our soluble systemic mediator results indicate a hyperactivated immune response related with patients that go on to fail response to therapy.

Of note, correlations between TILs and systemic soluble mediators highlighted differences between R and NR patients, suggesting a role of Treg, Th1, Th2, and Th17 cells in the treatment response mechanism. Conversely, in NR patients, CD68+ TAMs were correlated with CD4+ and PD-L1+ TILs, which may indicate macrophages characterized by the co-expression of CD4 and PD-L1, a previously described unconventional functional state of macrophages^78,79^. This state can lead to T-cell anergy, alteration of surface marker expression and cytokine secretion by these cells, M2 polarization, as well as proliferation, survival, and activation^80,81^, ultimately contributing to an immunosuppressive tumor environment and unfavorable clinical outcomes, as previously discussed. In support of the active inhibitory mechanism exhibited by PD-L1, we observed a strong negative correlation between PD-L1 expression in the TME and systemic levels of immune mediators related to anti-tumor responses via tissue migration and inflammation including IL-1a, IL-1b, IL-12, RANTES, SDF1a, TNF-a, TNF-b and others (Fig. 7).

Interestingly, positive correlations between CD8 and VEGF, as well as IL-6, were observed only in NR patients, indicating pathways involved in tumor growth in CC and T-cell infiltration and differentiation, which may collaborate with higher levels of CD8+ TILs in NR patients. This result reinforces a dysfunctional state of TILs with the involvement of PD-1/PD-L1 or PD-L2 interaction.

Finally, among both TILs and systemic soluble mediators, we identified independent biomarkers of chemotherapy efficacy for CC patients. Our study highlights the clinical relevance and prognostic value of PD-L1 and PD-L2 expression^49,82–84^. Additionally, growth factors (PDGFbb and FGF-basic), chemokines (IL-8 and IL-7), and proinflammatory cytokines (IL-12p40, IL-15, and TNF-α) were identified as promising potential biomarkers.

The limitations of our study include a modest sample size. As a prospective study, patients were enrolled over time based on who met the inclusion criteria. However, several statistically significant differences were found that form a network of immunoregulatory factors related to treatment response. Following enrollment, 90 patients were excluded from the study for a number of reasons including; 49 that did not complete all sessions of chemoradiotherapy and brachytherapy, 16 that were lost to follow-up, and 25 that were still in treatment at the end of the enrollment. Lastly, detailed specific sexual behavior (i.e. number of sexual partners or age at first sexual activity) was not included in the study due to difficulties in obtaining this type of information in an accurate manner.

In summary, our study provides new mechanistic insights into the dysregulation of the local and systemic immune response in NR patients. We also suggest biomarkers that may be feasible to incorporate into clinical practice upon further validation, which may improve treatment outcomes for CC patients.

## Methods

### Patient selection and clinical information

Initially, a total of 163 women with CC were enrolled in this prospective study. The eligibility criteria were primary diagnosis of CC, no preliminary cancer history, histology type; squamous cell carcinoma (SCC), or invasive cervical adenocarcinoma (ICA). However, 90 patients were excluded from the study, including 49 of them who did not complete all sessions of chemotherapy (paclitaxel + cisplatin) concurrent with radiotherapy and brachytherapy. Another group (16) was lost to follow-up and 25 were still in treatment. This left 73 volunteers that were included in this study from FIGO stages II and III. This study was approved by the Ethics Committee at Instituto Mario Penna under number 1.583.784, and the written informed consent was obtained from all patients between August 2017 and September 2020 at Mário Penna Institute (IMP), Belo Horizonte, Brazil.

The colposcopy punch biopsy (for histopathology and histochemical evaluation of immune cells) was taken from women with CC collected before the treatment began. The pathologist responsible for the Pathology Division at IMP performed histopathological analysis and diagnostic confirmation.

The clinical data and follow-ups were collected from medical records until twelve months after treatment. The patients were screened into two study groups, R and NR to chemoradiotherapy. Clinical response was evaluated by cervical and vaginal/abdominal pelvic examination, cytology, biopsy, and image exams (US, resonance, and tomography when required) before and after the end of treatment, according to FIGO (2015). R patients were considered those who had completely disappeared all local lesions in 4 months, followed by 8 and 12 months of evaluation after the end of treatment. The patients with partial response, residual or progressive disease in this period were considered NR patients.

### Follow-up data collection and missing data

The follow-up period extended from the first day of patient admission to the institution until the last visit, during August 2017 to September 2020. The OS was calculated by subtracting the first electronically collected hospital admission date by the last noted date. The results were presented in months of follow-up. The evaluation of the patient’s response (R and NR) was performed at 4, 8 and 12 months of follow-up. Losses of follow-up were not considered to the OS analysis. In this context, it was not possible to determine if some patients were alive (NR = 5 and R = 1). Consequently, missing values from these patients were excluded from the survival analysis. For other analyses, missing data were reported by the “n” value.

### TILs assessment on H&E-stained slides

Evaluation of TILs was done by following the guidelines for the assessment of TILs in solid tumors proposed by the International Immuno-Oncology Biomarker Working Group^15^. Blinded microscopy analyses of one section per patient (at 100 and 400× magnification) were performed. The semiquantitative analysis of the percentage of stromal TILs (ie, the area occupied by mononuclear inflammatory cells over the total stromal area) was a continuous variable tested in R and NR patients. The median was used as a cut-off to display low and high TILs densities.

### Histology and IHC

To investigate the cellular composition and immune status of the CC lesions, 69 samples (36 R and 33 NR patients) were submitted to IHC. Formalin-fixed paraffin-embedded (FFPE) blocks were cut at 4 µm thickness and sections were stained with H&E and IHC. For manual IHC, sections were incubated overnight at 4 °C with the following anti-human antibodies: anti-CD8, rabbit monoclonal (1:500, SP16, Cell Marque, Rocklin, USA); anti-CD4, rabbit monoclonal (1:200, SP35, Cell Marque, Rocklin, USA); anti-PD-1, mouse monoclonal (1:300, ab52587, clone NAT105, Abcam, Cambridge, MA); anti-PD-L1, rabbit monoclonal (1:500, ab205921, clone 28-8, Abcam, Cambridge, MA); anti-PD-L2, rabbit polyclonal (1:500, ab244332, Abcam, Cambridge, MA), anti-CD68, mouse monoclonal (1:100, ab955, Abcam, Cambridge, MA) and anti-FOXP3, rabbit polyclonal antibody (1:1000, PA1-16876, Thermo Fisher Scientific, Rockford, USA). The slides were stained with the Hidef Detection HRP Polymer System (Cell Marque, Rocklin, CA, USA) and visualized with 0.03% 3-3′-diaminobenzidine (SIGMA) in 0.01 M PBS, pH 7.4. A negative control without primary antibodies was generated for each sample.

### IHC scoring

The IR cells were evaluated in two separate compartments, stromal and intra-tumoral areas. The stromal compartment is delimited by parenchyma or conjunctive tissue and the intra-tumoral is delimited by tumor cell nests, where TILs are in direct cell–cell contact with carcinoma cells, and a third assessment was the total area, formed by the sum of stromal and intra-tumoral cell numbers (Supplementary Figs. 1a,d, 2a,d, 3a,d).

TILs expressing PD-1 were semi-quantitatively estimated due to the very few IR cell numbers. The percentage of TILs showing positive cytoplasmic membrane staining was determined using 5 fields at high power magnification in the total IR area. The density of cells and intensity of staining were determined on a scale of 0 to 3, with zero indicating < 5%, a score of 1 indicating 5–20%, a score of 2 indicating > 20–50%, and a score of 3 indicating > 50% of PD-1^+^ TILs^85^.

For the other markers, CD8, CD4, and FoxP3 (lymphocyte subpopulations), PD-L1 and PD-L2 (immune checkpoint molecules), and CD68+ TAMs, the absolute number of cells (TILs and TAMs) were quantified on one slide stained for each patient by digital microscopy images. For each slide patient, 5 randomly selected microscope fields were captured at 100× magnification (the total area analyzed was 15.7 mm^2^ per patient), using automated quantification in Image J software. The numbers of cells were counted in stromal and intra-tumoral areas separately using the "Particle analysis" function of Image J following optimization of pixel intensity, particle size, and circularity thresholds (https://imagej.net/Particle_Analysis). The medium of cells number was used for statistical tests. While the density of all cells, mononuclear and cancer cells expressing PD-L1 and PD-L2 was also determined by measuring the total IR area in stromal and intra-tumoral regions using the *Image J analysis software* (imagej.nih.gov/ij/). The evaluated area comprised 3 randomly selected fields using a light microscope NIKON at 100× magnification from a single histological section per patient. The average total area analyzed was 180 mm^2^ per patient. The criteria of positive cells and the delimitation of stromal and intra-tumoral regions were performed as described previously by our group^44^.

### Measurement of cytokine and other soluble mediators

Blood samples were collected in EDTA tube and processed for separation of plasma by centrifugation at 400×*g*, 10 min, 20 °C, and then stored at − 80 °C. For the assay, plasma samples (NR = 21, R = 26) were thawed and prepared by using the Bio-Plex Pro Human Cytokine Screening Panel, 48-Plex according to the manufacturer's instructions. Then, analytes were detected and quantified simultaneously on the Bio-Plex^®^ 200 system (BioRad Inc., USA), and all data was acquired using the Bio-Plex Manager software. Values are reported as pg/mL, based on standard curves provided by the kit.

### Statistical analysis

The data analysis provided a median and range for continuous variables and percentages for categorical variables. Clinical characteristics are compared by the Wilcoxon rank-sum test for continuous variables and Fisher exact tests for categorical variables. For immunohistochemistry and Bio-Plex data comparison, an unpaired two-tailed nonparametric Mann–Whitney test. The median value of the TILs in the stromal and intra-tumoral areas was used as a cutoff to stratify the CC patients into the low-expression group (≤ median) or the high-expression group (> median). Survival outcomes were assessed via Kaplan–Meier methods and compared using Log-rank tests. OS was defined as the period from the first day at the health service at IMP to the day of death from any cause or to the last date of observation. Correlation matrices were made using Spearman’s correlation. The OR with 95% CI were estimated using logistic regression in univariate analysis for each soluble mediator and cell marker. The predictive power of the model was evaluated by ROC curves with the Wilson/Brown method. The goodness of fit was carried out through of the Hosmer–Lemeshow test. Statistical analyses were conducted using RStudio version 4.1.3 and GraphPad Prism 8.4 (GraphPad Software Inc., La Jolla, Ca, USA). The criteria for statistical significance were set up as *P ≤ 0.05, **P ≤ 0.001, and ***P ≤ 0.0001.

All methods were carried out in accordance with relevant guidelines and regulations.

### Supplementary Information

Supplementary Information.

## Data Availability

All the data supporting the findings of this study are available within the article and its supplementary information files and from the corresponding author upon reasonable request.

## Abbreviations

CC: Cervical cancer
R: Responder
NR: Non-responder
TILs: Tumor-infiltrating lymphocytes
TAM: Tumor-associated macrophages
IR: Immunoreactive
HPV: Human papillomavirus
FIGO: International Federation of Gynecology and Obstetrics
PD-1: Programmed cell death protein 1
PD-L1: Programmed death-ligand 1
PD-L2: Programmed death-ligand 2
FoxP3: Forkhead Box protein 3
OR: Odds ratio
OS: Overall survival
N: Lymph node invasion
M: Metastasis
CI: Confidence interval
SCC: Squamous cell carcinoma
ICA: Invasive cervical adenocarcinoma
H&E: Hematoxylin & Eosin
IHC: Immunohistochemistry
TME: Tumor microenvironment
ROC: Receiver operating characteristic
AUC: Area under the curve
PFS: Progression-free survival
ICA: Invasive cervical adenocarcinoma
IMP: Mário Penna Institute
FFPE: Formalin-fixed paraffin-embedded
