# Supplementary material for: Linking tumor immune infiltrate and systemic immune mediators to treatment response and prognosis in advanced cervical cancer
Source: Sci Rep. 2023 Dec 19;13:22634. doi: 10.1038/s41598-023-49441-2 (PMC10730812; doi:10.1038/s41598-023-49441-2)
Supplement: Supplementary file 1 — Supplementary Information. [file 41598_2023_49441_MOESM1_ESM.pdf]

SUP Figure 1

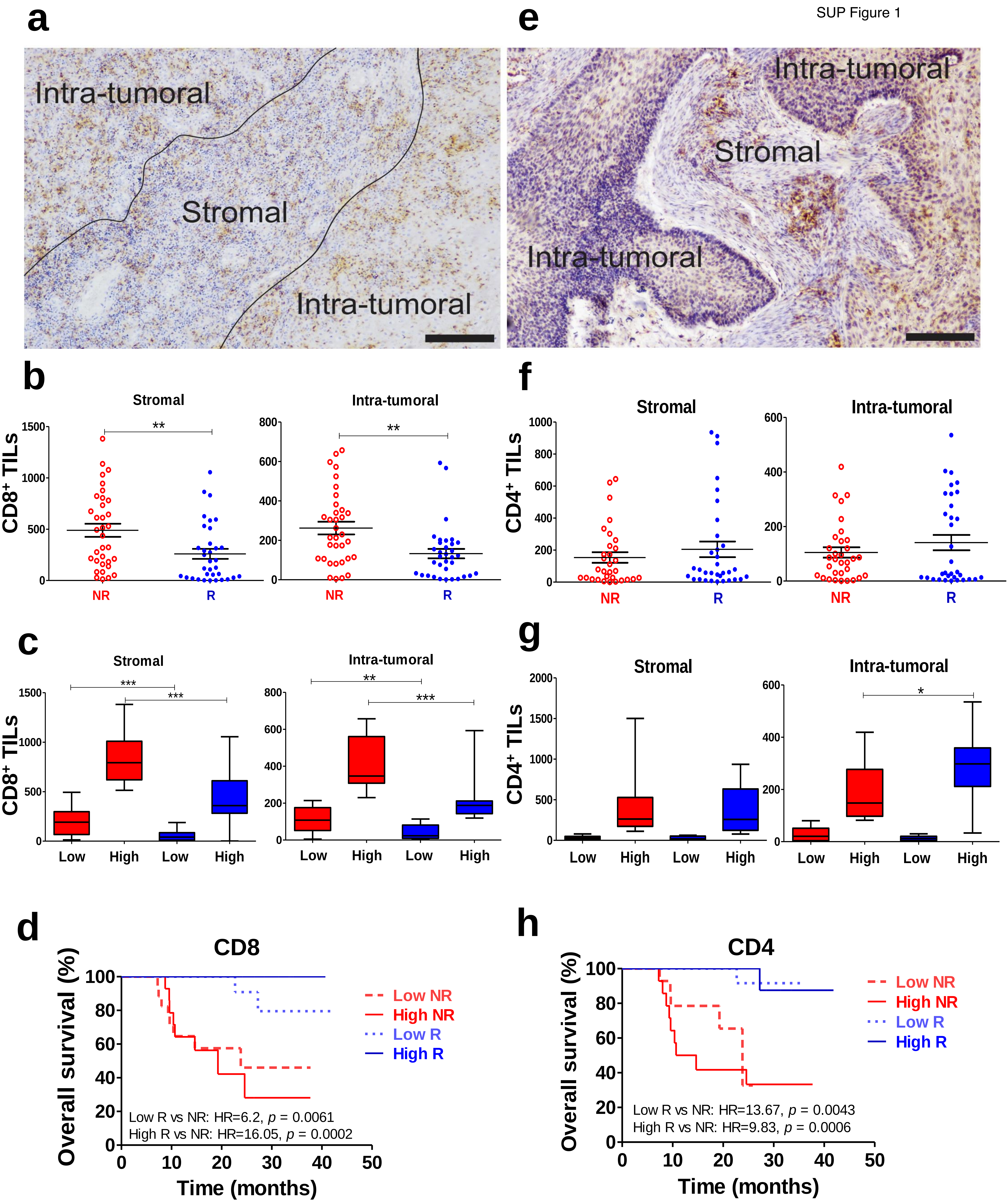
a
Intra-tumoral
Stromal
Intra-tumoral
e
Intra-tumoral
Stromal
Intra-tumoral
b
Stromal
Intra-tumoral
CD8+ TILs
NR
R
f
Stromal
Intra-tumoral
CD4+ TILs
NR
R
c
Stromal
Intra-tumoral
CD8+ TILs
Low
High
Low
High
g
Stromal
Intra-tumoral
CD4+ TILs
Low
High
Low
High
d
CD8
Overall survival (%)
Time (months)
Low NR
High NR
Low R
High R
Low R vs NR: HR=6.2, p = 0.0061
High R vs NR: HR=16.05, p = 0.0002
h
CD4
Overall survival (%)
Time (months)
Low NR
High NR
Low R
High R
Low R vs NR: HR=13.67, p = 0.0043
High R vs NR: HR=9.83, p = 0.0006

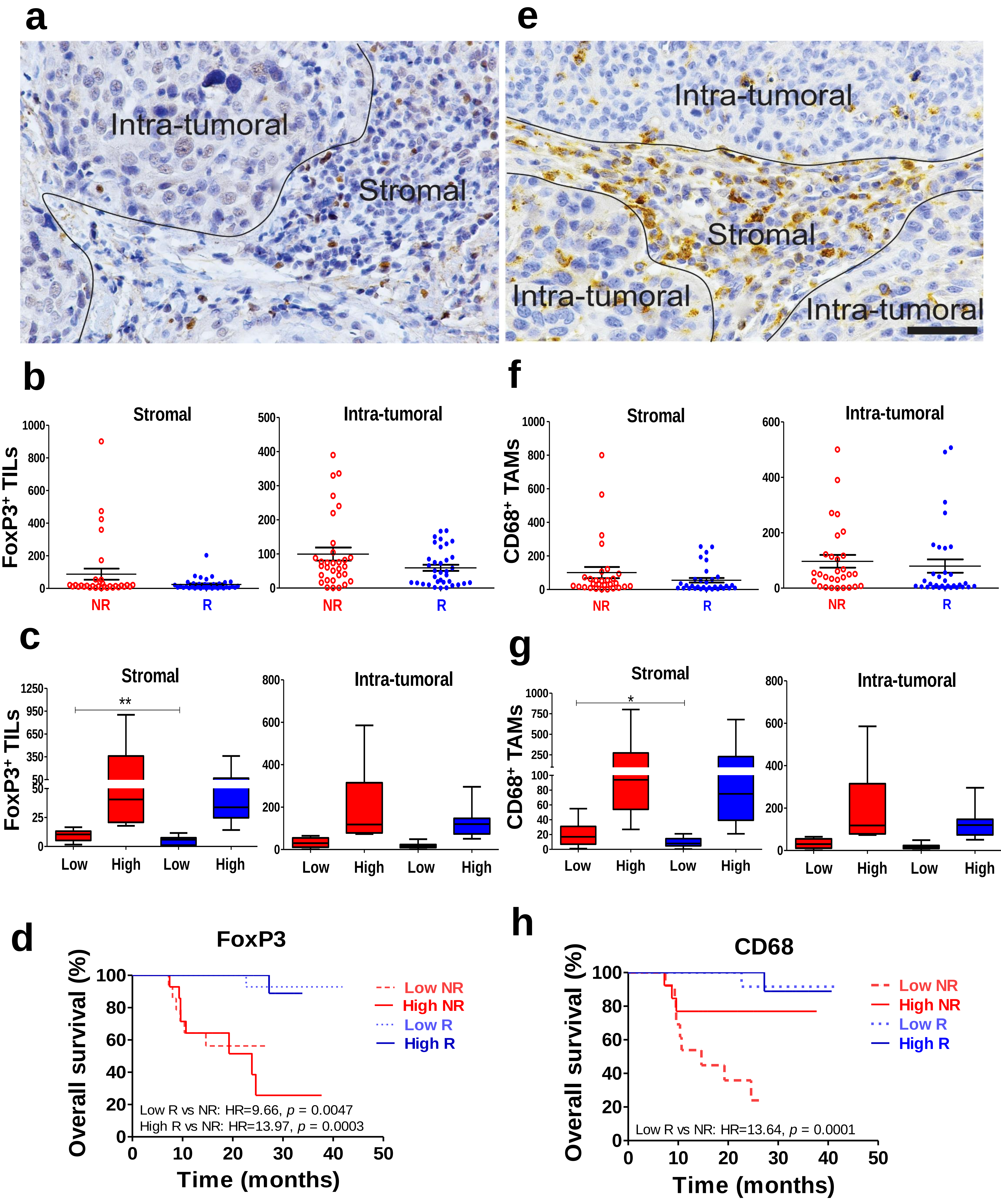
a
Intra-tumoral
Stromal
e
Intra-tumoral
Stromal
Intra-tumoral
Intra-tumoral
b
Stromal
Intra-tumoral
FoxP3+ TILs
NR
R
f
Stromal
Intra-tumoral
CD68+ TAMs
NR
R
c
Stromal
Intra-tumoral
FoxP3+ TILs
**
Low
High
Low
High
g
Stromal
Intra-tumoral
CD68+ TAMs
*
Low
High
Low
High
d
FoxP3
Overall survival (%)
Time (months)
Low NR
High NR
Low R
High R
Low R vs NR: HR=9.66, p = 0.0047
High R vs NR: HR=13.97, p = 0.0003
h
CD68
Overall survival (%)
Time (months)
Low NR
High NR
Low R
High R
Low R vs NR: HR=13.64, p = 0.0001

SUP Figure 3

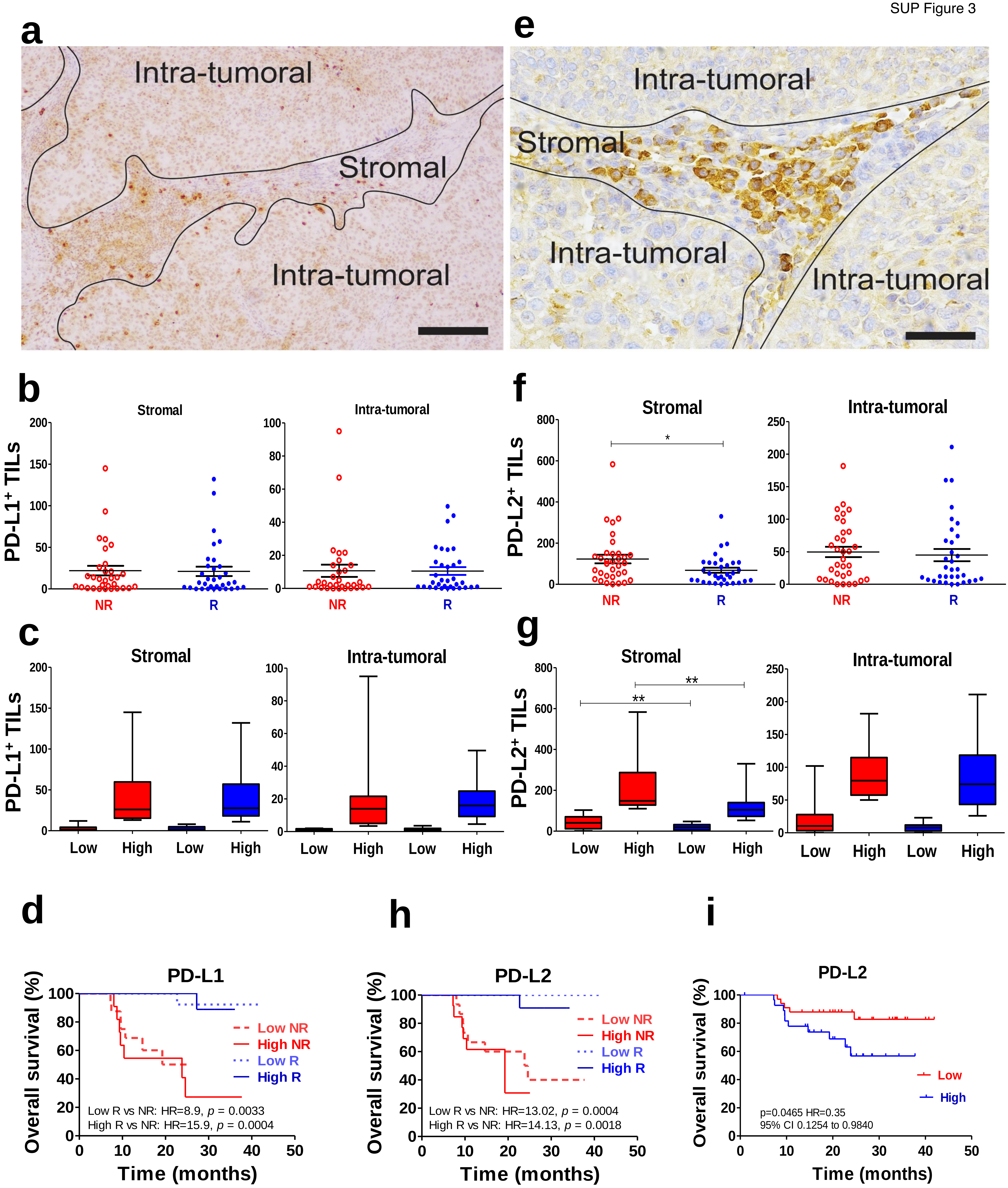
a
Intra-tumoral
Stromal
Intra-tumoral
e
Intra-tumoral
Stromal
Intra-tumoral
Intra-tumoral
b
Stromal
Intra-tumoral
PD-L1+ TILs
NR
R
f
Stromal
Intra-tumoral
PD-L2+ TILs
NR
R
c
Stromal
Intra-tumoral
PD-L1+ TILs
Low
High
g
Stromal
Intra-tumoral
PD-L2+ TILs
Low
High
d
PD-L1
Overall survival (%)
Time (months)
Low NR
High NR
Low R
High R
Low R vs NR: HR=8.9, p = 0.0033
High R vs NR: HR=15.9, p = 0.0004
h
PD-L2
Overall survival (%)
Time (months)
Low NR
High NR
Low R
High R
Low R vs NR: HR=13.02, p = 0.0004
High R vs NR: HR=14.13, p = 0.0018
i
PD-L2
Overall survival (%)
Time (months)
Low
High
p=0.0465 HR=0.35
95% CI 0.1254 to 0.9840

SUP Figure 4

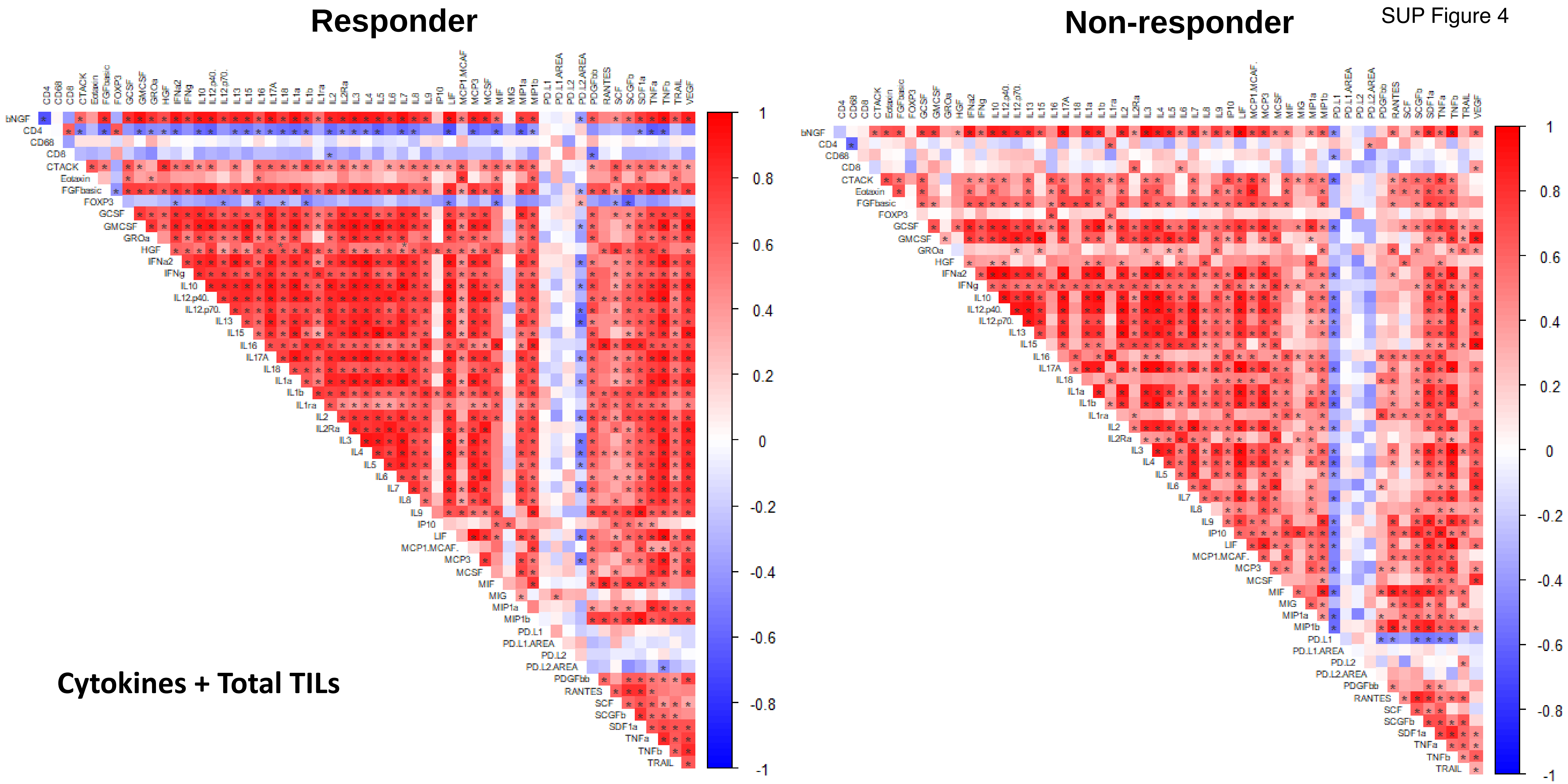
Responder
Non-responder
Cytokines + Total TILs
bNGF
CD4
CD68
CD8
CTACK
Eotaxin
FGFbasic
FOXP3
GCSF
GMCSF
GROa
HGF
IFNa2
IFNg
IL10
IL12.p40.
IL12.p70.
IL13
IL15
IL16
IL17A
IL18
IL1a
IL1b
IL1ra
IL2
IL2Ra
IL3
IL4
IL5
IL6
IL7
IL8
IL9
IP10
LIF
MCP1.MCAF.
MCP3
MCSF
MIF
MIG
MIP1a
MIP1b
PD.L1
PD.L1.AREA
PD.L2
PD.L2.AREA
PDGFbb
RANTES
SCF
SCGFb
SDF1a
TNFa
TNFb
TRAIL
VEGF
1
0.8
0.6
0.4
0.2
0
-0.2
-0.4
-0.6
-0.8
-1

SUP Figure 5

Responder

Non-responder

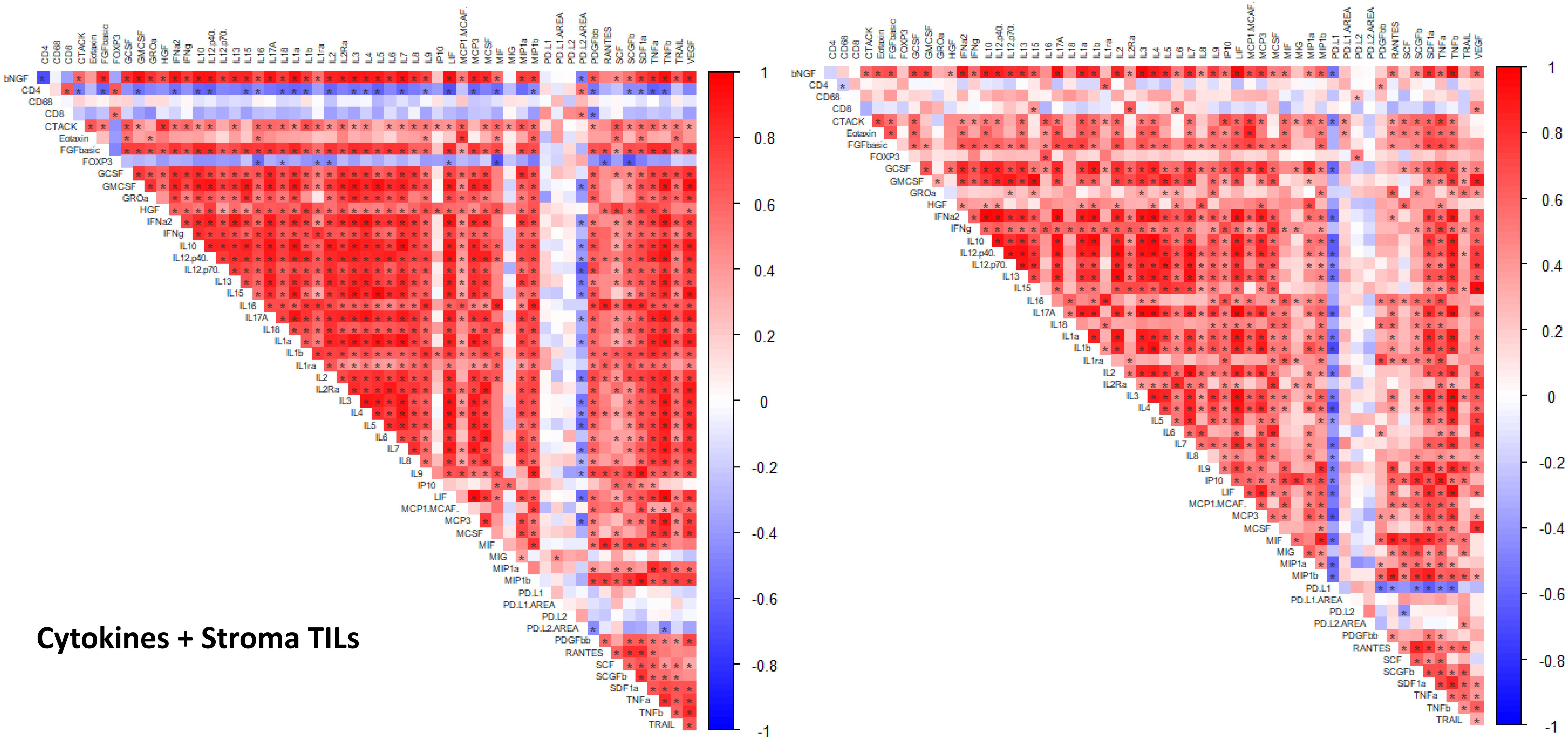

SUP Figure 1

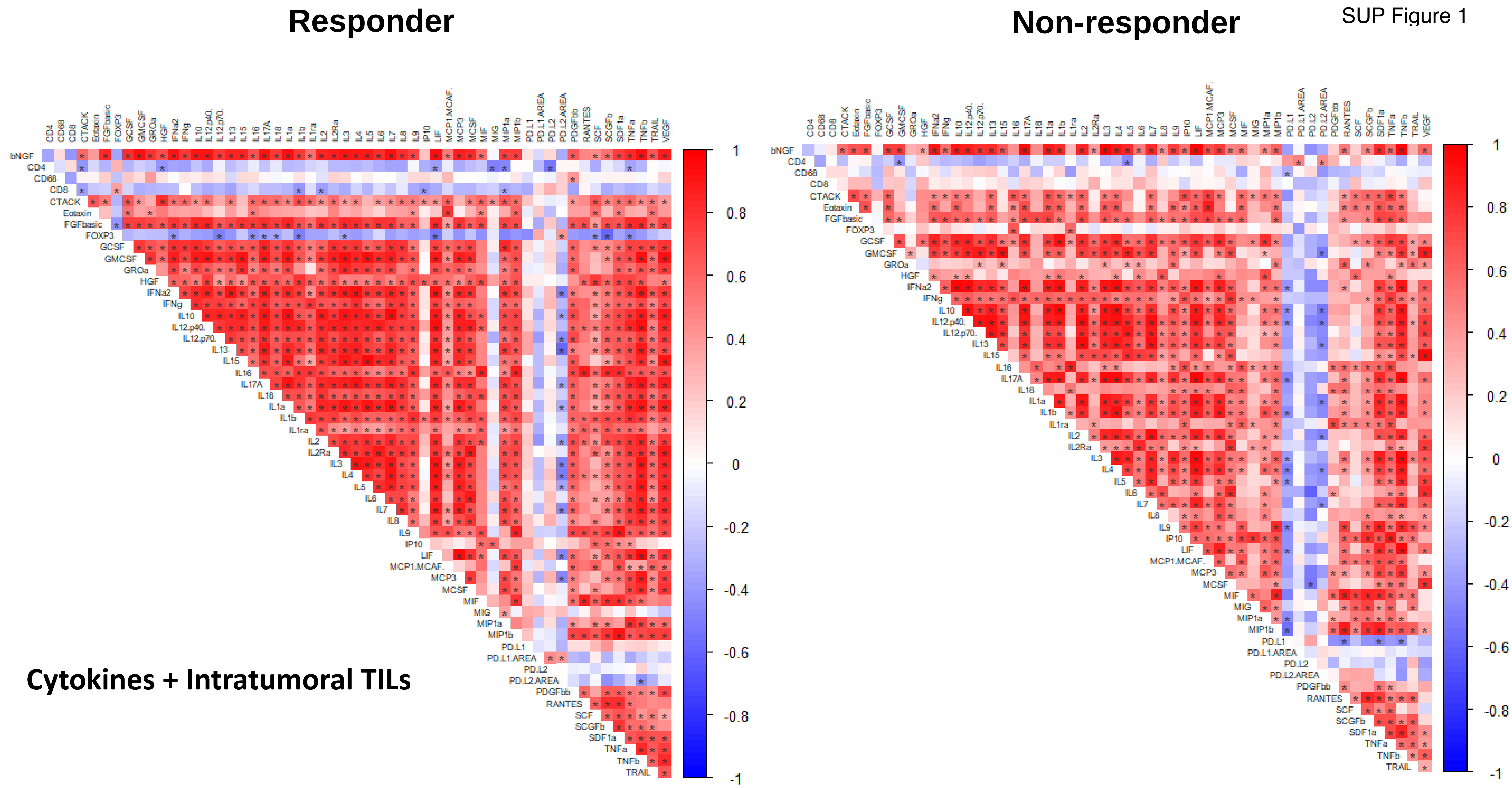

Supplementary Figure 1. NR and R CC patients presented higher $CD8^+$ and higher $CD4^+$ TILs respectively, when analyzed in stromal and intratumoral compartments. (**a** and **e**) Representative IHC images of $CD8^+$ **(a)** and $CD4^+$ **(e)** TILs distributed in stromal and intra-tumoral regions. **(a)** Scale bar = 200μm. **(e)** Scale bar = 50μm. (**b** and **f)** Morphometric analysis showed NR patients with a higher number of $CD8^+$ TILs than in R patients (**b**, NR n=33, R n=34) and no difference in the number of $CD4^+$ TILs (**f**, NR n=32, R n=33) in stromal and intra-tumoral regions. (**c**) Number of $CD8^+$ TILs plotted in both regions (low R vs. NR and high R vs. NR, $p < 0.001$; NR low, n=16, NR high, n=17; R low, n=17 and R high, n=17). (**g**) Higher number of $CD4^+$ TILs in the intra-tumoral region of R (high R vs. NR; NR low, n=16; NR high, n=15; R low, n=16 and R high, n=15). (**d** and **h**) Kaplan-Meier survival curves show a comparison of OS between low and high infiltration of $CD8^+$ and $CD4^+$, *p* values determined using the log-rank test. **(d)** Survival analysis of CC patients showed an association between a low level of $CD8^+$TILs in R vs. NR (p=0.0061) and a high $CD8^+$ R vs. NR (p=0.0002; NR low, n=21, NR high, n=15; R low, n=17 and R high, n=17). (**h**) Survival analysis of CC patients showed an association between a low level of $CD4^+$TILs in R vs. NR (p=0.0043) and a high $CD4^+$ R vs. NR (p=0.0006; NR low, n=16, NR high, n=15; R low, n=16 and R high, n=15). Statistical differences are indicated by asterisks (*) $p \leq 0.05$, (**) $p \leq 0.01$, and (***) $p \leq 0.001$. The *p* values were calculated using the Mann-Whitney test. CC, Cervical Cancer; NR, Non-responder (Red) patients; R, Responder (Blue) patients.

Supplementary Figure 2. NR CC patients presented a high number of stromal FoxP3+TILs and stromal CD68+ TAMs when analyzed in low and high densities. (**a** and **e**) Representative IHC images of $FoxP3^+$ TILs (**a**) and $CD68^+$ TAMs (**e**) distributed in stromal and intra-tumoral regions. Scale bar = 50μm. Morphometric analysis did not show a difference between the groups (**b**, NR, n=32; R, n=36 and **f,** NR, n=30; R, n=34). (**c**) Higher number of $FoxP3^+$ TILs (low R vs. NR $p \leq 0.01$; NR low, n=16; NR high, n=16; R low, n=19 and R high, n=17), and (**g**) $CD68^+$ TAMs (low R vs. NR $p \leq 0.05$; NR low, n=15, NR high, n=15; R low, n=19 and R high, n=15) in the stromal region of NR patients. (**d**) Survival analysis of CC patients showed association between low level of $FoxP3^+$TILs in R vs. NR (p=0.0047) and high $FoxP3^+$ R vs. NR (p=0.0003; NR low, n=16, NR high, n=15; R low, n=18 and R high, n=18). (**h**) Survival analysis of CC patients showed association between low level of $CD68^+$TAMs in R vs. NR (p=0.0001; NR low, n=16, NR high, n=15; R low, n=18 and R high, n=18). Statistical differences are indicated by asterisks (*) $p \leq 0.05$, (**) $p \leq 0.01$, and (***) $p \leq 0.001$. The *p* values were calculated using the Mann-Whitney test. CC, Cervical Cancer; NR, Non-responder (Red) patients; R, Responder (Blue) patients.

Supplementary Figure 3. NR CC patients presented a high number of stromal PD-$L2^+$TILs. (**a** and **e**) Representative IHC images of PD-$L1^+$ (**a**) and PD-$L2^+$ TILs (**e**) distributed in stromal and intra-tumoral regions. (**a**) Scale bar = 200μm. (**e**) Scale bar = 50μm. Morphometric analysis did not present a difference in the number of PD-$L1^+$ TILs (**b**, NR, n=31; R n=34 and **c**, NR, n=17; NR high, n=14; R low, n=16 and R high, n=18). NR presented a higher number of PD-$L2^+$ TILs than in R patients (**f**, NR, n=33, R n=34, $p \leq 0.05$) and when compared low (R vs. NR, $p \leq 0.01$) and high (R vs. NR, $p \leq 0.01$) number of TILs in the stromal region (**g,** NR low, n=17; NR high, n=16; R low, n=17 and R high, n=17). (**d)** OS analysis revealed an association between a low level of PD-$L1^+$ TILs in R vs. NR (p=0.0033) and a high PD-$L1^+$ R vs. NR (p=0.0004; NR low, n=17, NR high, n=14; R low, n=17 and R high, n=17). **(h)** Survival analysis of CC patients showed an association between a low level of PD-$L2^+$ TILs in R vs. NR (p=0.0004) and a high PD-$L2^+$ R vs. NR (p=0.0018; NR low, n=18, NR high, n=15; R low, n=17 and R high, n=17). Statistical differences are indicated by asterisks (*) $p \leq 0.05$, (**) $p \leq 0.01$, and (***) $p \leq 0.001$.

The *p* values were calculated using the Mann-Whitney test. CC, Cervical Cancer; NR, Non-responder (Red) patients; R, Responder (Blue) patients.

Supplementary Figure 4. Correlation matrix of all 48 cytokines and Total TILs (stromal with intratumoral). Evaluation in the R and NR groups. NR, Non-responder (n=21); R, Responder (n = 26). All statistical analyses were performed with Spearman's correlation Test (*$p \leq 0.05$).

Supplementary Figure 5. Correlation matrix of all 48 cytokines and Stroma TILs. Evaluation in the R and NR groups. NR, Non-responder (n=21); R, Responder (n = 26). All statistical analyses were performed with Spearman's correlation Test (*$p \leq 0.05$).

Supplementary Figure 6. Correlation matrix of all 48 cytokines and intratumoral TILs. Evaluation in the R and NR groups. NR, Non-responder (n=21); R, Responder (n = 26). All statistical analyses were performed with Spearman's correlation Test (*$p \leq 0.05$).

Graphic Abstract. Mechanistic insights and biomarkers identified in locally advanced CC patients evaluated before chemoradiation therapy. With all results taken together, NR patients would present the highest levels of $CD8^+$ TILs that collaborate with positive correlations between CD8 and VEGF, and IL-6 as well as the highest levels of these systemic soluble immune mediators. According to the literature, this scenario could promote tumor growth in CC patients as well as proliferation and invasion of $CD8^+$ T cells. However, a dysfunctional state of TILs would be generated by PD-1/PD-L1 or PD-L2 interaction, since PD-1 scores were associated with NR outcome and PD-L1 IR area were highest in this group. In addition, immunosuppressive or unconventional macrophages expressing both CD4 and/or PD-L1 would participate in therapeutic failure perhaps by inducing T cell anergy, M2 polarization, and alterations in the cytokine's secretion. In contrast, R patients would have functional $CD8^+$ TILs considering the correlations with IL-2 and PDGFbb. It is also suggested that after proper cooperation of different polarization profiles of $CD4^+$ TILs, these cells are regulated by *Treg*. In addition, NR patients have cytokine levels closer to healthy individuals. All of this contributes to healthy tissue recovery. Finally, our findings also indicate promising biomarkers candidates that predict response to conventional treatment in CC. CC, Cervical Cancer; NR, Non-responder; R, Responder patients.

Supplementary Table 1. List of cutoff values for all cell markers.

Marker	Group	Total Sum Cutoff	Stromal Cutoff	Intra-tumoral Cutoff
CD8	NR (n=33)	754.0	493.0	214.0
	R (n=34)	334.0	156.5	116.5
CD4	NR (n=32)	282.1	74.3	81.0
	R (n=33)	261.8	62.0	30.0
Foxp3	NR (n=32)	86.8	15.8	69.0
	R (n=36)	82.2	12.9	47.3
CD68	NR (n=30)	107.0	35.5	50.5
	R (n=34)	48.5	21.0	16.0
PD-L1	NR (n=31)	15.0	12.0	2.0
	R (n=34)	9.7	7.6	3.6
PD-L2	NR (n=33)	169.0	103.2	38.4
	R (n=34)	68.5	47.4	22.4

The median cell density for each marker, in the stromal and intra-tumoral regions. These median values were subsequently used as cutoff values for low and high cell densities.
